# Disentangling Self-Atomic Motions in Polyisobutylene by Molecular Dynamics Simulations

**DOI:** 10.3390/polym13040670

**Published:** 2021-02-23

**Authors:** Yasmin Khairy, Fernando Alvarez, Arantxa Arbe, Juan Colmenero

**Affiliations:** 1 Physics Department, Faculty of Science, Zagazig University, Zagazig 44519, Egypt; yasmin_ph@yahoo.com; 2 Centro de Física de Materiales (CSIC, UPV/EHU), Paseo Manuel de Lardizabal 5, E-20018 San Sebastián, Spain; fernando.alvarez@ehu.eus (F.A.); a.arbe@ehu.eus (A.A.); 3 Materials Physics Center MPC, Paseo Manuel de Lardizabal 5, E-20018 San Sebastián, Spain; 4 Departamento de Polímeros y Materiales Avanzados: Física, Química y Tecnología (UPV/EHU), Apartado 1072, E-20080 San Sebastián, Spain; 5 Donostia International Physics Center (DIPC), Paseo Manuel de Lardizabal 4, E-20018 San Sebastián, Spain

**Keywords:** dynamics of polymers, fully atomistic molecular dynamics simulations

## Abstract

We present fully atomistic molecular dynamics simulations on polyisobutylene (PIB) in a wide temperature range above the glass transition. The cell is validated by direct comparison of magnitudes computed from the simulation and measured by neutron scattering on protonated samples reported in previous works. Once the reliability of the simulation is assured, we exploit the information in the atomic trajectories to characterize the dynamics of the different kinds of atoms in PIB. All of them, including main-chain carbons, show a crossover from Gaussian to non-Gaussian behavior in the intermediate scattering function that can be described in terms of the anomalous jump diffusion model. The full characterization of the methyl-group hydrogen motions requires accounting for rotational motions. We show that the usually assumed statistically independence of rotational and segmental motions fails in this case. We apply the rotational rate distribution model to correlation functions calculated for the relative positions of methyl-group hydrogens with respect to the carbon atom at which they are linked. The contributions to the vibrational density of states are also discussed. We conclude that methyl-group rotations are coupled with the main-chain dynamics. Finally, we revise in the light of the simulations the hypothesis and conclusions made in previously reported neutron scattering investigations on protonated samples trying to address the origin of the dielectric β-process.

## 1. Introduction

During the last decades, neutron scattering (NS) has proved to be an extremely valuable microscopic technique to decipher the structural and dynamical properties of glass-forming systems, including polymeric materials [1,2,3]. In particular, its sensitivity to hydrogen motions allows characterizing the molecular dynamics of protonated systems at length scales in the Å to nm range. At such scales, well above the glass-transition temperature $T_{g}$ the structural or α-relaxation is the most relevant dynamical process. In the early 90’s, by using backscattering techniques on protonated samples [poly(vinyl methyl ether) (PVME), phenoxy (PH) and poly(vinyl chloride) (PVC)], the correlation function corresponding to self-atomic (hydrogen) motions in this regime was characterized by stretched exponentials with similar stretching exponents (β-parameter) as those obtained by dielectric relaxation for the α-process [4]. The momentum transfer (*Q*) dependence of the characteristic times was found to be correlated with the shape parameter β such that the atomic probability distributions could be approximately described by Gaussian functions. The underlying motions were qualified as anomalous diffusion-like processes where the atomic mean squared displacement $\langle r^{2}\rangle$ increases sublinearly with time ($\langle r^{2}\rangle \propto t^{\beta }$). These results were later confirmed by similar experiments on ’canonical’ polymers [like e.g., 1,4-polybutadiene (PB), poly(vinyl ethylene) (PVE), polyisoprene (PI) and polyisobutylene (PIB)] [5], and also on other systems like ionic liquids [6,7], alcohols [8], nanocomposites [9,10], hydration water [11] or other complex macromolecules like lignin [12]. Backscattering measurements are however restricted to a rather limited dynamic window and the results are affected by the instrumental resolution function, which hampers an accurate determination of the spectral shape. Deconvolution from the instrumental resolution is possible by the neutron spin echo (NSE) technique, which in addition accesses the widest dynamic range for NS instruments. The first NSE results on a fully protonated polymer were reported in Ref. [13]. The sample was PIB. Though still subjected to uncertainties in the determination of the exact value of the shape parameter β, the NSE data revealed unambiguously stretched functional forms and *Q*-dependences of the characteristic times compatible with the Gaussian approximation, confirming the previous general conclusions. In the high *Q*-range investigated, however, indications for deviations from Gaussian behavior were found, suggesting a crossover from Gaussian to non-Gaussian behavior in a *Q*-range of about 1 ${Å}^{-1}$.

Such deviations were investigated in more detail on PI with deuterated methyl groups by combining NS (backscattering and NSE) experiments with molecular dynamics (MD) simulations [14,15]. This combined approach results to be an extremely powerful tool, as it has been shown in a series of systems during the last years (see, e.g., [3,16]). To explain the behavior found for the hydrogen motions in PI, the so-called anomalous jump diffusion model was proposed. This model is a generalization of the well-known jump-diffusion model [17] to the case of sublinear diffusion. Similar crossover from Gaussian to non-Gaussian behavior was also later reported for other glass-forming polymers like PB [18], PVE [18,19], PVME [20], poly(methyl methacrylate) (PMMA) [21], poly(ethylene propylene) (PEP) [22], head-to-head polypropylene (hh-PP) [22], poly(vinyl acetate) (PVAc) [23], poly(vinyl pirrolidone) (PVP) [24], poly(ethyl methacrylate) (PEMA) [25] and poly(tetrahydrofurane) (PTHF) [26], as well as for other systems as e.g., n-alkanes [27] or H-bonding liquids [28,29,30]. The anomalous jump diffusion model has also been applied to some of these polymers and seems to be successful in capturing the essence of the caging effect occurring in these systems in a very simple way. The limited *Q*-range of the NSE experiments on PIB [13] prevented however a reliable application of such model.

In addition to the α-relaxation, other processes like the β-relaxation might be found in polymers. In two cases, PB and PIB, there were attempts to characterize by NS the molecular motions behind the β-process. This is an extremely difficult task, since in the relatively high frequency window accessed by NS, contributions not only from the β-process but also from the α-relaxation are expected to be present. For PB, coherent scattering data obtained on a deuterated sample were analyzed assuming a scenario of statistically independent α and β-processes [31,32]. The localized motions behind the β-relaxation could be characterized as rotational jumps of the chain building blocks around their center of mass. This was corroborated by MD-simulations some years later [33]. Such an approach was also applied to describe the NS results on PIB. From the description of the self-correlation function accessed on a protonated sample, the length of the jumps involved in the localized motions giving rise to the dielectric β-process was determined to be of about 2.7 Å [34]. However, NSE experiments on a fully deuterated sample addressing collective relaxation in PIB and evaluated in the same framework delivered a much smaller value (0.5–0.9 Å) for such jump distance [35]. Conversely, the dielectrically detected β-relaxation showed very similar features as the so-called δ-process reported from NMR experiments [36,37,38], that was interpreted as rotations of methyl groups (which are not dielectrically active). Interestingly, the NS spectra of PIB in the vicinity of $T_{g}$ do not show any clear hint of classical hopping of its methyl groups [39,40], as it is usually the case in glass-forming polymers [41]. The NS experimental signature of methyl group dynamics in PIB is the presence of a pronounced, broad and structured peak in the vibrational density of states (VDOS) measured on fully protonated samples [39,42]. Such data in fact reveal the total-hydrogen averaged VDOS, containing contributions from both, methyl-group and main-chain hydrogens. The reported peak is located at an energy of about 40 meV, i.e., a rather high value as compared to the typical energies corresponding to the methyl-group librational peaks in polymers [41]. This observation was explained in Ref. [39] in terms of a strong coupling between the methyl groups, which also may be related to the strong steric hindrance of the polymer chain in PIB. Adams et al. [42] proposed that the peak at ≈40 meV was due to a broadened methyl group torsional frequency, and the structure observed at 25 K (a kind of shoulder or additional peak at higher frequencies) was due mainly to the C-C-C skeletal vibrations being in the same region of the spectrum. They remarked the importance of using selective deuteration to experimentally resolve possible different components of the structured broad peak observed.

Polyisobutylene is thus one of the glass-forming polymers that has been most extensively investigated by NS [13,34,35,39,40,42,43,44,45,46,47,48,49,50,51,52,53,54]. At the same time, however, there are still a number of open questions regarding the interpretation of these results. In addition, we cannot forget that PIB is a very interesting polymer also for industrial applications due to its well-known and unusual physical properties; remarkably low $T_{g}\;\approx$ 200 K resulting in low cost processability, high packing efficiency and low gas permeability. Any contribution to the microscopic understanding of the underlying dynamical processes in this system is always important.

With these ideas in mind, we have performed fully atomistic MD-simulations on PIB in a wide temperature range above $T_{g}$. We have followed the strategy that has proved to be highly successful in previous studies that combined NS and MD-simulations [3,16]. First, the cell is validated by direct comparison of magnitudes computed from the simulation and measured by NS. We addressed problems related with collective features in a previous work [55]. There, the realism of the simulated cell was checked by comparison of data corresponding to the static and dynamic structure factor. Here we focus on self atomic motions. Consistently, the validation will be performed against NS results on protonated samples mainly determined by the self-correlation function of the hydrogens. Once the reliability of the simulated cell is assured, with all the information contained in the simulations at hand, we can ask questions that an experimentalist cannot answer due to technical limitations. In particular, we can address how good are the approximations involved in the experimental data analysis (always a crucial problem).

This work is focused on the characterization of the dynamics of the different kinds of atoms in PIB. In theory, partial deuteration of the sample allows selectively investigating by NS the motions of the remaining hydrogens. However, partially deuterated PIB samples are not easy to synthesize and consequently there are no available NS results that allow isolating main-chain from methyl-group hydrogen motions. Moreover, since the incoherent scattering cross-section of carbon atoms is 0, their self-motions are inherently inaccessible by NS. One particularly interesting question is how different are the motions of the total subensamble of hydrogens from those of the main-chain atoms. Do the latter also show deviations from Gaussian behavior? Along this paper, we first discuss the dynamic heterogeneity associated to the different atomic species and the applicability of the anomalous jump diffusion model to the different kinds of atoms in PIB. Thereafter, the characterization of the methyl-group hydrogen motions is performed. We note that these motions are very commonly found to contribute the the quasielastic spectra together with other processes like the segmental relaxation (see, e.g., [56,57,58,59,60,61,62]) and these contributions are not easy to disentangle. We show that the usually assumed statistically independence of rotational and segmental motions is inappropriate for PIB. Exploiting the possibility offered by the simulations of calculating correlation functions corresponding to relative motions of atoms (including those defined in real space), we apply the rotational rate distribution model and discuss the particularities of PIB methyl-group dynamics. The contributions to the VDOS are also disentangled. Finally, we revise in the light of the simulations the hypotheses and conclusions made in previously reported neutron scattering investigations on protonated samples trying to address the origin of the dielectric β-process of this polymer.

## 2. Molecular Dynamics Simulations

Fully atomistic molecular dynamics simulations were carried out using the COMPASS forcefield implemented within the commercial software package MATERIALS STUDIO 5.1. The cubic cell contained 20 PIB chains of 70 monomers ($M_{w}$ = 3923 g/mol, i.e., smaller than the entanglement mass $\;M_{e}\;\approx$ 7000 g/mol [63], and a total number of atoms *N* = 16,840). The system was simulated at the temperatures of 470, 390, 365, 335 and 320 K, following the procedure explained in Ref. [55], to carry out 100 ns dynamics.

### Computed Magnitudes

The trajectory in space of a single atom is described by the self part of the van Hove correlation function $G_{s}\left(\vec{r},t\right)$. $G_{s}\left(\vec{r},t\right)$ is the probability to find an atom at time *t* at a position $\vec{r}$ if it was at $\vec{r}$ = 0 for *t* = 0:

(1) $$G_{s}\left(\vec{r},t\right)=\frac{1}{N}\langle \sum_{i=1}^{N}\delta \left[\vec{r}-\left({\vec{r}}_{i}\left(t\right)-{\vec{r}}_{i}\left(0\right)\right)\right]\rangle .$$

Here ${\vec{r}}_{i}$ is the position of the atom *i* and *N* the number of nuclei in the sample. The brackets denote the ensemble average. The Fourier transform of $G_{s}\left(\vec{r},t\right)$ in $\vec{Q}$-space is the so-called intermediate scattering function $F_{s}\left(\vec{Q},t\right)$,

(2) $$F_{s}\left(\vec{Q},t\right)=\int G_{s}\left(\vec{r},t\right)\exp\left(i\vec{Q}\vec{r}\right)d\vec{r}.\;$$

For some simple cases—free nuclei in a gas, harmonic crystals, simple diffusion at long times; $G_{s}\left(\vec{r},t\right)$ is a Gaussian function [64,65]; in an isotropic system this implies

(3) $$G_{s}^{gauss}\left(r,t\right)={\left[\frac{\alpha (t)}{\pi }\right]}^{3/2}\exp\left[-\alpha \left(t\right)r^{2}\right].$$

In the Gaussian approximation the even moments of $G_{s}\left(r,t\right)$

(4) $$\langle \;r^{2n}\;\rangle =\int_{0}^{\infty }r^{2n}G_{s}\left(r,t\right)4\pi r^{2}dr$$

can be straightforwardly calculated. For instance, the mean squared displacement of the atom $\langle \;r^{2}\left(t\right)\;\rangle$ is given by $\langle \;r^{2}\left(t\right)\;\rangle =3/\left[2\alpha \left(t\right)\right]$. Moreover, in such Gaussian case $F_{s}\left(Q,t\right)$ is entirely determined by $\langle \;r^{2}\left(t\right)\;\rangle$:

(5) $$F_{s}^{gauss}\left(Q,t\right)=\exp\left[-\frac{\langle r^{2}\left(t\right)\rangle }{6}Q^{2}\right].\;$$

In more general cases, deviations of $G_{s}\left(r,t\right)$ from the Gaussian form [Equation ((3)] may be expected. $F_{s}\left(Q,t\right)$ can then be expressed in terms of its expansion in *Q* (see, e.g., Ref. [66])

(6) $$F_{s}\left(Q,t\right)=\exp\left[-\frac{\langle \;r^{2}\left(t\right)\;\rangle }{6}Q^{2}+\frac{\alpha_{2}\left(t\right){\langle \;r^{2}\left(t\right)\;\rangle }^{2}}{72}Q^{4}+\ldots \right]$$

where $\alpha_{2}\left(t\right)$ giving the leading correction is the so called second order non-Gaussian parameter. It is defined as [67]

(7) $$\alpha_{2}\left(t\right)=\frac{3}{5}\frac{\langle \;r^{4}\left(t\right)\;\rangle }{{\langle \;r^{2}\left(t\right)\;\rangle }^{2}}-1.$$

Evidently, in the Gaussian case $\alpha_{2}\left(t\right)$ = 0.

## 3. Validation

As we will compare MD-simulation with experimental neutron scattering (NS) results, we will introduce some magnitudes and functions related with NS that will be invoked. In general the neutron intensity scattered by a sample contains an incoherent contribution reflecting the self motion of atoms, as above defined, and a coherent contribution related to the atomic pair correlations. These contributions are weighted by the factors $I_{inc}$ and $I_{coh}\left(Q\right)$ respectively. These factors depend on the incoherent and coherent scattering lengths $b_{i}^{inc}$ and $b_{i}^{coh}$ of the nuclei in the sample (*i* refers to the nucleus considered) as:

(8) $$I_{inc}=\frac{1}{N}\sum_{i}^{N}{\left(b_{i}^{inc}\right)}^{2}$$

and

(9) $$I_{coh}\left(Q\right)=\frac{1}{N}\langle \sum_{i}^{N}\sum_{j}^{N}b_{i}^{coh}b_{j}^{coh}e^{i\vec{Q}\left({\vec{r}}_{i}-{\vec{r}}_{j}\right)}\rangle .$$

Here, ${\vec{r}}_{i}$ and ${\vec{r}}_{j}$ are taken at the same time and the brackets mean the thermal average.

Hydrogen presents a very large value for the incoherent scattering length ($b_{H}^{inc}$ = 25.274 fm). In a fully protonated polymer sample consisting of H and C, the scattered intensity is completely dominated by the incoherent contribution from the protons (C only scatters coherently with $b_{C}^{coh}$ = 6.6511 fm, and $b_{H}^{coh}$ = −3.7406 fm). Therefore, in such case $I_{inc}\;>>\;I_{coh}\left(Q\right)$ for all *Q*-values and NS results are predominantly determined by $F_{s}\left(Q,t\right)$ related to the self motion of the hydrogens.

In this work, to validate the simulated cell we have used previously published neutron spin echo (NSE) results on fully hydrogenated PIB [13]. NSE is distinguished from all other dynamic neutron scattering techniques in that it measures time-dependent correlation functions. The NSE signal is given by [68,69]:

(10) $$S_{NSE}\left(Q,t\right)=\frac{I_{coh}\left(Q\right)\tilde{F}\left(Q,t\right)-\frac{1}{3}I_{inc}F_{s}\left(Q,t\right)}{I_{coh}\left(Q\right)-\frac{1}{3}I_{inc}}$$

where $\tilde{F}\left(Q,t\right)$ and $F_{s}\left(Q,t\right)$ are the intermediate pair correlation function (normalized to its value at *t* = 0) and the self correlation function above defined respectively.

**Figure 1:**
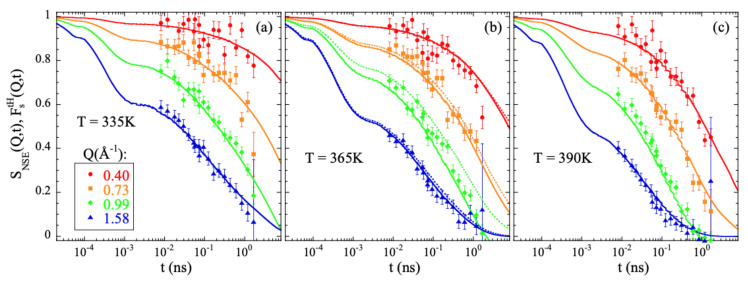
Comparison of the neutron spin echo results obtained from measurements on a fully hydrogenated PIB sample [13] (symbols) and calculated from the simulations (solid lines) at 335 K (**a**), 365 K (**b**) and 390 K (**c**) at the *Q*-values indicated in (**a**). Bandpass corrections have been applied to the experimental data (see, e.g., [19]). The value considered for the band pass time was 0.2 ps. For comparison, the $F_{s}\left(Q,t\right)$ calculated for all hydrogens at 365 K is shown by the dotted lines in (**b**).

Figure 1 shows the comparison between the experimental results measured by NSE on a fully hydrogenated sample and computed from the atomic trajectories in the simulation following Equation (10). The agreement is excellent. The simulations fairly reproduce the *Q*- and *T*-dependence of the characteristic times and spectral shape experimentally observed. It is noteworthy that no shift in timescale has been imposed for a direct matching.

**Figure 2:**
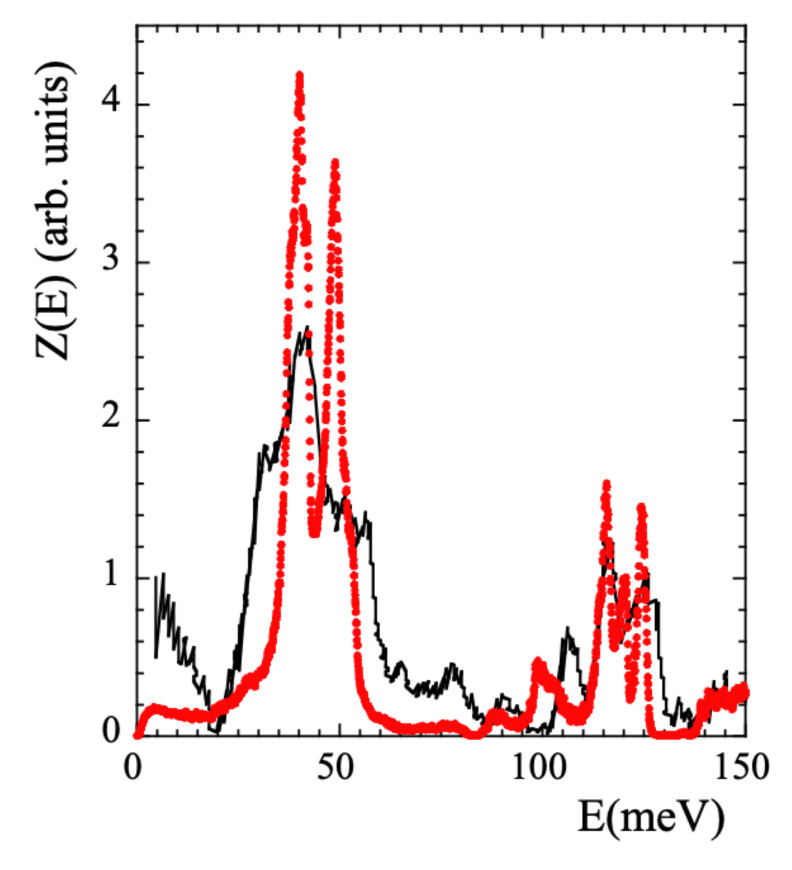
Comparison of the VDOS obtained from measurements on a fully hydrogenated PIB sample [42] (black line) and calculated from the simulations (red circles) at 25 K.

Though the main focus of this work is on the dynamical behavior at times longer than the picosecond, we will also discuss some aspects related with the vibrational processes. Therefore, we have also considered the vibrational density of states (VDOS, Z(E)) to validate the simulated cell. The VDOS of PIB was investigated applying NS to fully protonated samples (revealing the VDOS corresponding to all the hydrogens in the system) by Frick et al. [39] at 240 K and later by Adams et al. [42] at 25 K, using better resolution. These data are reproduced in Figure 2 in the region below 150 meV.

From the simulations, the Z(E)-function can be calculated from the spectral density of the velocity autocorrelation function as

(11) $$Z\left(E\right)\;\propto \;\int_{-\infty }^{+\infty }e^{-iEt}\langle \vec{v}\left(0\right)\vec{v}\left(t\right)\rangle dt$$

where the velocity autocorrelation function is calculated in terms of the velocity autocorrelation function of each (Hydrogen) atom as:

(12) $$\langle \vec{v}\left(0\right)\vec{v}\left(t\right)\rangle =\frac{1}{N}\sum_{i}\langle \vec{v_{i}}\left(0\right)\vec{v_{i}}\left(t\right)\rangle$$

The Z(E) was calculated running a NVT dynamics of 800 ps at 25 K. However, we note that equilibrating a simulated cell at 25 K is just impossible. Thus the dynamics was run at 25 K but in the cell previously equilibrated at 320 K, i.e., with the density and the structure corresponding to 320 K. Data were recorded every 0.01 ps. The comparison with the results of Ref. [42] is presented in Figure 2. The overall agreement found is rather good, supporting the realism of the simulations also regarding vibrational aspects. We note that there are no quantum mechanic corrections in MD-simulations, which should mainly affect the high-energy region of the spectrum. Therefore, the comparison has to be taken with care in such a region.

## 4. Results and Discussion

Once the cell has been properly validated, from the MD-trajectories it is possible to calculate also magnitudes that are not easily (or not at all) experimentally accessible, like e.g., correlation functions in real space, or analyzing separately the different kinds of nuclei (including the ’experimentally invisible’ carbons) in the sample. This information can be of utmost help to interpret experimental observations, and/or to check different theoretical frameworks. Such capabilities of the simulations are exploited in this section.

### 4.1. Distinguishing the Different Atomic Species: Chemical Dynamic Heterogeneity in PIB

**Figure 3:**
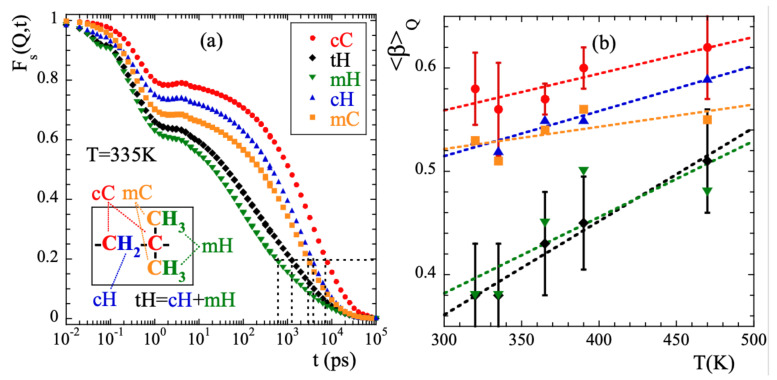
(**a**) Intermediate scattering function calculated for the different kinds of atoms in PIB at *Q* = 1.5 ${Å}^{-1}$ and 335 K. Horizontal dotted line shows reference level for the definition of $\tau_{0.2}$. Inset: Scheme of the PIB monomer illustrating the nomenclature for the different kinds of atoms considered. (**b**) Temperature dependence of the *Q*-averaged values of the stretching parameter for the different species. Symbols as in (**a**). For the cases of cC and tH, the vertical bars show the range over which the *Q*-dependent values span at each temperature. Lines are linear regression fits to guide the eye.

For a *Q*-value of 1.5 ${Å}^{-1}$ and *T* = 335 K, Figure 3a shows the intermediate incoherent scattering function of the different kinds of atoms in PIB: main-chain carbons (cC), main-chain hydrogens (cH), methyl-group carbons (mC) and methyl-group hydrogens (mH) (see inset in Figure 3a for the definitions). With tH we denote the total hydrogens (main-chain and methyl-group hydrogens). Figure 3a reveals that: (i) for any type of atom the scattering function decays in two main steps, before and after ≈1 ps; (ii) the curves completely decay to 0 in the dynamic window investigated in this temperature range. The achievement of such a full decorrelation can be attributed to the dynamics of the α-relaxation; (iii) the atomic motions in PIB are very heterogeneous. Defining a global characteristic time $\tau_{0.2}$ as that at which the function reaches the value of 0.2 (see dotted line in the figure), at 335 K we find a difference of more than one order of magnitude between $\tau_{0.2}^{mH}$ and $\tau_{0.2}^{cC}$. (iv) the decay at short times is more pronounced for methyl-group atoms than for main-chain atoms, and (v) the stretching of the second decay is much stronger for mH than for the other atoms in the system. Finally, as expected, the behavior of tH is very close to that of mH –6 out of the 8 hydrogens in PIB are located in the methyl groups.

We have characterized the second decay (times longer than 2 ps) of the incoherent scattering functions of the different atomic species by using the commonly invoked stretched exponential or Kohlrausch-Williams-Watts functional form [1]:

(13) $$F_{s}\left(Q,t\right)=A\exp\left[-{\left(\frac{t}{\tau_{w}\left(Q,T\right)}\right)}^{\beta }\right].$$

**Figure 4:**
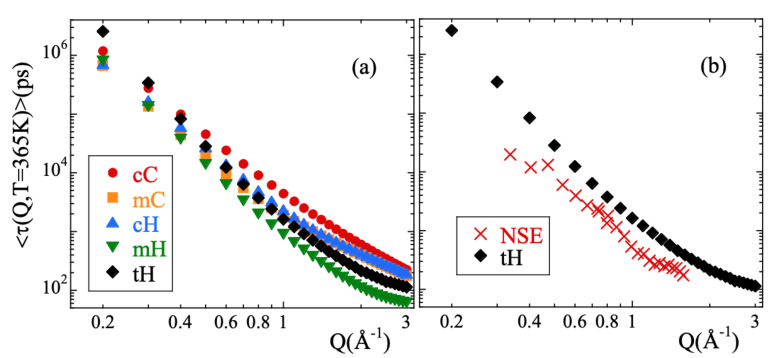
Momentum transfer dependence of the average characteristic times obtained at 365 K for (**a**) the different atomic species and (**b**) for the total hydrogens compared with the experimental NSE results reported in Ref. [13].

Here *A* is the amplitude accounting for the decay of the correlations in the microscopic regime, 0<β≤1 is the stretching exponent characterizing the deviations with respect to a single exponential decay and $\tau_{w}\left(Q,T\right)$ is the characteristic time. In a first step, we fitted Equation (13) to the simulated curves allowing the three parameters to float. For a given species and temperature, we did not find a systematic variation with *Q* of the values obtained for the β-parameter. Therefore, we calculated the *Q*-averaged values of this parameter ${\langle \beta \rangle }_{Q}$ and considered them as representative for each temperature and kind of atom. They are presented in Figure 3b. The highest values of this parameter are found for cC and the lowest for mH (and tH). A tendency to increase with increasing temperature is observed for all the species, in particular for mH. In a second step, we fitted again Equation (13) by fixing the values of the β-parameters to the average values ${\langle \beta \rangle }_{Q}$. The descriptions obtained were reasonably good. Given the change of the shape parameter with temperature and considered species, in the following we will discuss the results corresponding to the characteristic times in terms of the average characteristic times 〈τ〉, that in the case of KWW functions are related with $\tau_{w}$ through the expression $\langle \tau \rangle =\Gamma \left(1/\beta \right)\tau_{w}/\beta$. For 365 K, Figure 4 shows the *Q*-dependence of 〈τ〉 obtained for the different species. In the low-*Q* limit (large length scales), all the times roughly coincide. With increasing *Q* they decrease, showing a certain curvature in the log-log representation. The values corresponding to cC, mC and cH tend to coincide in the high-*Q* range (local length scales) where those of mH remain faster.

**Figure 5:**
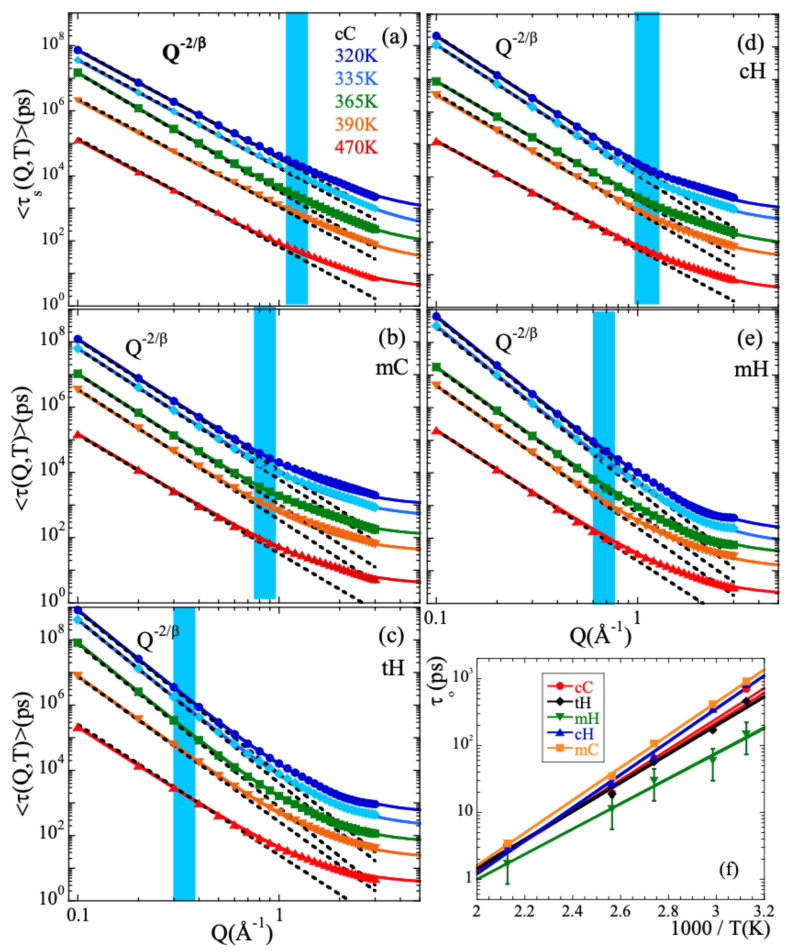
(**a**–**e**) Momentum transfer dependence of the average characteristic times obtained for the different temperatures (see color code in Panel (**a**)) for the different atomic species: main-chain carbons (**a**); methyl-group carbons (**b**); total hydrogens (**c**); main-chain hydrogens (**d**); methyl-group hydrogens (**e**). Continuous lines are fits of the anomalous jump diffusion model [Equation (15)]. Dotted lines show the asymptotic Gaussian law [Equation (14)]. In both cases, the values of ${\langle \beta \rangle }_{Q}$ shown in Figure 3b have been used. Shadowed areas indicate the *Q*-range where deviations from the Gaussian approximation start to develop. (**f**) Temperature dependence of the elementary characteristic time involved in the anomalous jump diffusion model for the different kinds of atoms in PIB. Representative error bars are shown for the mH species. Solid lines are fits of Arrhenius equations with activation energy $E_{a}^{AJD}$ (see Table 1).

Figure 5a–e show the *Q*-dependence of the average characteristic times for the different temperatures investigated. Each panel corresponds to a given atomic species. In the low-*Q* region, in all cases the data follow well a power law increase as

(14) $$\tau \;\propto \;Q^{-2/\beta }$$

(see dotted lines). Here, the β-value used at each temperature is the *Q*-averaged value shown in Figure 3b. This observation implies the Gaussian form of the scattering function (Equation (5) in such a *Q*-range [4], as can be deduced when Equation (14) is considered together with Equation (13). Note that since the value of the stretching parameter used in the fits is common for all *Q*-values, considering $\tau_{w}$ or 〈τ〉 is equivalent for the discussion of the *Q*-dependence of the characteristic times. The only difference is a prefactor.

The asymptotic Gaussian laws $\tau \;\propto \;Q^{-2/\beta }$ cease to describe the *Q*-dependence of the characteristic times in a *Q*-range that varies from one species to the other but for a given kind of atom hardly depends on temperature (see shadowed areas in Figure 5). At higher *Q*-values, the *Q*-dependence is weaker than that given by Equation (14), indicating deviations from the Gaussian approximation. Thus, the crossover from Gaussian to non-Gaussian behavior is found not only for the total hydrogens (as it was reported from the NSE experiments [13]), but for all kinds of atoms in PIB.

The anomalous jump diffusion model [14,15] has been applied to the different atomic species distinguished in PIB. In this model, an atom remains in a given site for a time $\tau_{o}$, where it vibrates around a center of equilibrium. After $\tau_{o}$, it moves rapidly to a new position. These jumps are assumed to occur with random orientations and their moduli *ℓ* are distributed according to a function $f_{o}\left(\ell \right)=\frac{\ell }{\ell_{o}^{2}}\exp\left(-\frac{\ell }{\ell_{o}}\right)$ which involves a preferred jump distance $\ell_{o}$. The characteristic time then follows the law

(15) $$\tau_{w}=\tau_{o}{\left(1+\frac{1}{\ell_{o}^{2}Q^{2}}\right)}^{\frac{1}{\beta }}$$

Figure 5 shows as continuous lines the very satisfactory descriptions obtained. Again, we have imposed here the *Q*-averaged values of β shown in Figure 3b. For the different types of atoms, the temperature variation of $\ell_{o}$ and $\tau_{o}$ are represented in Figure S1 of the Supplemental Material (SM) and Figure 5f respectively. The values of $\ell_{o}$ do not appreciably change with temperature. The $\ell_{o}$-values averaged over all the temperatures investigated are listed in Table 1. For the main-chain atoms, they increase from cC to cH (0.3 to 0.4 Å, respectively); hydrogens undergo larger jumps than carbons in the decaging process. The value obtained for cH is comparable to that reported for the main-chain hydrogens in e.g., polyisoprene (0.42 Å) [14,15]. For methyl-group atoms this length is larger than for the main-chain counterparts and rather similar for both mH and mC (0.50 and 0.54 Å). For the total hydrogens, the value obtained for $\ell_{o}$ is largest. This result can be understood considering that in this case the *Q*-dependence of the characteristic times reflects deviations from Gaussian behavior which are a consequence not only of the discrete nature of the underlying mechanism for sublinear diffusion (basic ingredient of the anomalous jump diffusion model) but also of the heterogeneous dynamics developed by cH and mH. The more pronounced deviations from Gaussian behavior are captured in this simple model by an effectively larger value of the jump distance. A similar situation was discussed for PVE [19], PVME [20], styrene-butadiene-rubber (SBR) and polystyrene (PS) [70]. Additional ingredients were also included to describe the behavior found in complex systems like e.g., confined liquid crystals [71] or proton diffusion in polymer eletrolyte fuel cell membranes [72]. Furthermore, this intrinsic heterogeneity –together with the occurrence of mH-rotations as we will show in the following section– is also the cause of large stretching for tH. On the other hand, the values of $\tau_{o}$ corresponding to cC, cH and mC are rather similar and show nearly identical values of the activation energy $E_{a}^{AJD}$ (see Table 1). For mH the obtained values are smaller and the activation energy weaker. Thus, methyl-group rotations lead to a quicker delocalization of mH atoms.

It is noteworthy that in essence the anomalous jump diffusion model incorporates the ingredient of caging by considering a distribution of jumps underlying the diffusive-like motion of atoms in the α process. The cage effect is the main concept involved also in the mode coupling theory (MCT) [73,74]. The applicability of MCT to the case of PIB was explored in a previous work [75]. The localization length $r_{sc}$ involved in the MCT was found to be $r_{sc}$ = 0.30 Å for cC and $r_{sc}$ = 0.50 Å for mH. These values are very similar to those obtained in the anomalous jump diffusion model for the preferred jump distance. We note that this was also the case for cH in PI [15].

From the simulations we thus confirm the experimental observation of stretching of $F_{s}\left(Q,t\right)$, its Gaussian behavior at low *Q*s and deviations at higher *Q*-values reported from experiments. The larger window covered, the better statistics of the data and the capability of isolating the incoherent contribution has made it possible to determine with more accuracy the value of the shape parameter and the limits of the Gaussian approximation. It is meaningful to compare the outcome of the analysis of the NSE results [13] and those of the simulations. In the interpretation of the NSE data, it is assumed that $S_{NSE}\left(Q,t\right)=F_{s}^{tH}\left(Q,t\right)$. Figure 1b shows that this is a very good approximation, though the coherent contribution can be noticeable in the *Q*-range of the first structure factor peak (≈$1\;{Å}^{-1}$). Figure 4b compares the average relaxation times for tH from the simulations with those reported from the experiments. The latter are systematically faster. This is mainly due to the choice of the shape parameter β: the NSE results were fitted by assuming a fixed value of 0.55 deduced from the analysis of collective data. This value is sensitively larger than that obtained from the simulations (see Figure 3b). Such relatively small values of β are due to the heterogeneous motions of cH and mH, i.e., ultimately to the occurrence of methyl-group rotations. These motions are discussed in more detail in the following.

### 4.2. Methyl Group Dynamics

In the high temperature range here investigated, the mH are expected to undergo classical hopping in a rotational potential. We have found indications for the rotations in the low values of the β-parameter used to describe the second decay of $F_{s}\left(Q,t\right)$ for mH and in the faster corresponding times observed in the high *Q*-range with respect to those of the other species. However, since the segmental dynamics (α-relaxation) is superimposed, a two-steps decay of $F_{s}\left(Q,t\right)$ after the microscopic regime is not observed for mH in the whole temperature range investigated (see e.g., Figure 3a), preventing a direct characterization of these rotational motions.

**Figure 6:**
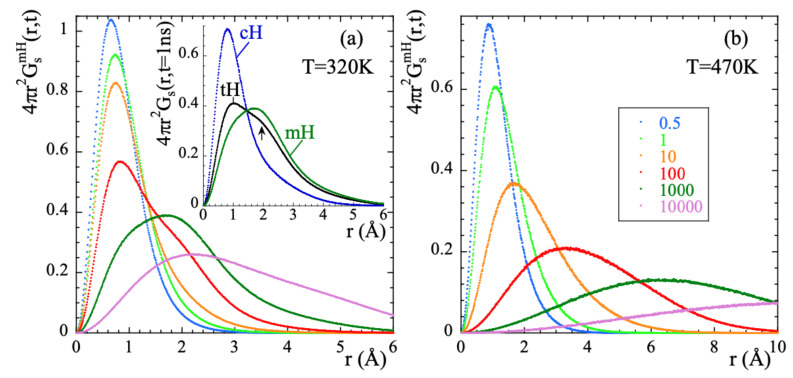
Radial probability distribution function calculated for methyl-group hydrogens at 320 K (**a**) and 470 K (**b**) at the different times (in ps) indicated in (**b**). For *t* = 1000 ps, the insert in (**a**) compares this function for methyl-group hydrogens, main-chain hydrogens and total hydrogens. The arrow marks the location of a second peak (hump) in the function corresponding to tHs (see the text).

Exploring the correlation functions directly in real space (the self-part of the van Hove correlation functions $G_{s}\left(r,t\right)$) can be of utmost help to characterize the atomic motions. For the two extreme temperatures here investigated, Figure 6 represents these functions for mH at different times. At 470 K, the features of the probability distribution function correspond to a diffusive-like process: increasing broadening and shift toward larger distances with increasing time. At 320 K some hints of a jump-like process with the appropriate characteristic length ($r_{HH}$ = 1.78 Å is the distance between the hydrogens in the methyl group) could be envisaged in the range 100…1000 ps. However, the overall behavior is also rather close to a diffusional process.

**Figure 7:**
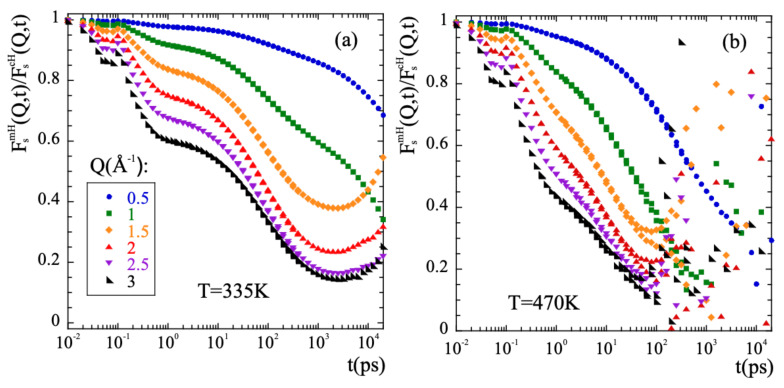
Result of dividing the intermediate scattering functions corresponding to methyl-group hydrogens and main-chain hydrogens at the *Q*-values indicated in (**a**) for *T* = 335 K (**a**) and *T* = 470 K (**b**).

Thus, in order to characterize the methyl-group rotations the data have to be ’cleaned out’ from the diffusive-like motions of the α-relaxation. In a first approximation, it could be assumed that both kinds of motions occur simultaneously and are statistically independent. We have checked this hypothesis for PIB. Under such conditions, the incoherent scattering functions for methyl group hydrogens $F_{s}^{mH}\left(Q,t\right)$ would be given by the product of the incoherent scattering functions corresponding to the purely rotational motion and to the α-relaxation process. The latter can be approximated with the incoherent scattering function calculated for main-chain hydrogens $F_{s}^{cH}\left(Q,t\right)$. This approach was successfully used in the case of PI [76] and is in fact inspired in the way an experimentalist would face this kind of problem, provided the availability of NS measurements on fully protonated and partially deuterated samples. This allows to obtain in a straightforward way the deconvoluted rotational function by simple division of the two known functions, $F_{s}^{Deconv}\left(Q,t\right)=F_{s}^{mH}\left(Q,t\right)/F_{s}^{cH}\left(Q,t\right)$. If the invoked assumptions are correct, consistent results should be obtained. First of all, its functional form should be that of a localized motion. This implies that its long-time limit should be a constant value, the so-called Elastic Incoherent Incoherent Structure Factor (EISF). The EISF gives account for the geometry of the motion. Figure 7 shows the results of applying the deconvolution approach to PIB. A pronounced first decay in the microscopic regime below 1 ps is seen, that is due to the larger motional amplitude at shorter times of the mH with respect to the cH. A second decay towards a plateau is clearly visible at high *Q*-values for the low temperature (Figure 7a), that is reached at about 2 ns. At longer times, mainly in the low-*Q* range, the obtained curves do not really show a constant value, as it should happen if motions are independent. At the highest temperature of 470 K (Figure 7b), no plateau can even be identified in the curves. We can thus infer that the initial assumption of statistical independence fails. If the segmental contribution is approximated by considering the scattering function of main-chain carbons instead of that of main-chain hydrogens, the deconvolution does not work better. The conclusion is that the deconvolution assumption implying the statistical independence of α-process and methyl-group rotations fails for PIB, not only for long times and high temperatures, but also at the lowest temperatures investigated at intermediate times and intermolecular length scales, as we show in detail in the SM.

**Figure 8:**
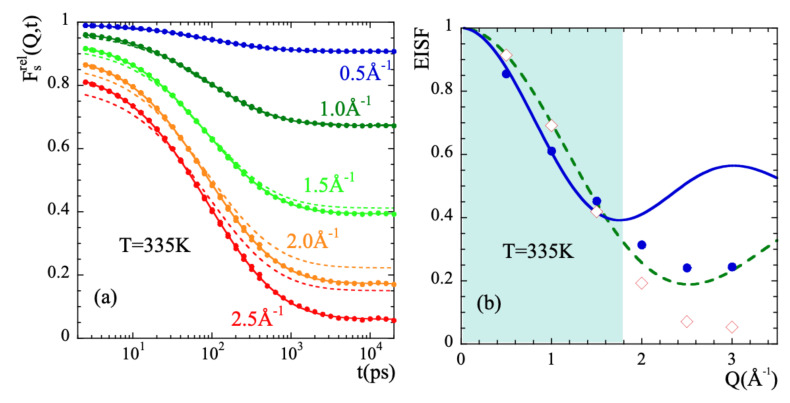
(**a**) Intermediate scattering function of methyl-group hydrogens corresponding to their motion relative to the methyl-group carbon $F_{s}^{rel}\left(Q,t\right)$ at the *Q*-values indicated. The dotted lines are fits with the RRDM imposing the theoretical 3-fold EISF (dotted line in (**b**)) and *Q*-independent σ and $\tau_{o}^{MG}$, while in the solid lines free A, EISF, σ and $\tau_{o}^{MG}$ have been allowed. The such resulting EISF values are shown in (**b**) (diamonds). There, the ‘apparent EISF’ obtained from the deconvolution approach (see the text) is shown by the solid circles. The continuous line is a description of these data within the experimental window explored in Ref. [34] (shadowed area) in terms of Equation (18). The resulting *d*-value is 2.6 Å. In all cases the temperature is 335 K.

**Figure 9:**
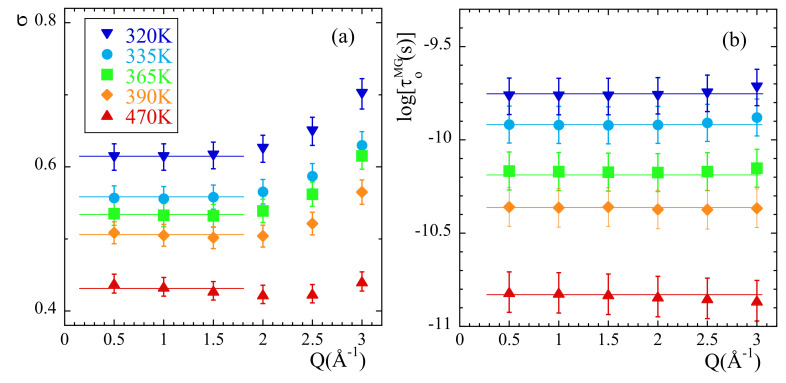
Momentum transfer dependence of the RRDM parameters σ (**a**) and $\tau_{o}^{MG}$ (**b**) obtained from the fit of $F_{s}^{rel}\left(Q,t\right)$ at the temperatures indicated in (**a**). Lines show the values considered as representative for these parameters.

In order to characterize the rotational dynamics of the methyl groups in PIB, we have calculated from the simulations the correlation functions corresponding to the relative motions of mH with respect to the mC to which they are linked. Figure 8a shows as an example the intermediate scattering function $F_{s}^{rel}\left(Q,t\right)$ calculated at 335 K and different *Q*-values. We applied the rotational rate distribution model (RRDM) [41,77,78] (see SM) to describe this function. The RRDM assumes the presence of a Gaussian distribution of potential barriers for methyl group rotations as consequence of the disorder. The only free parameters are the width σ and the center $\tau_{o}^{MG}$ of the log-normal distribution function of characteristic times for rotation H(logτ). In addition, obviously, an amplitude *A* accounting for the microscopic contribution has to be used. The RRDM with *Q*-independent $\tau_{o}^{MG}$ and σ parameters provides the descriptions shown in Figure 8a as dotted lines. They are very good for *Q*-values in the range $Q\;\leq \;1.5\;{Å}^{-1}$ and show some deviations at higher *Q*-values; in particular, the theoretical EISF:

(16) $$EISF=\frac{1}{3}\left(1+2\frac{\sin\left(Qr_{HH}\right)}{Qr_{HH}}\right).$$

seems to underestimate the values obtained from the simulations. Allowing free *A*, EISF, $\tau_{o}^{MG}$ and σ parameters the fits reproduced very well all the results (solid lines in the figure). Figure 8b shows the values obtained for the EISF parameter at 335 K (empty diamonds) as compared with the theoretical prediction Equation (16). Figure 9 displays $\tau_{o}^{MG}$ and σ for all temperatures investigated.

As can be seen in Figure 8b, the EISF-values show a more pronounced decay than that corresponding to a 3-fold potential for *Q*-values > 1.5$\;{Å}^{-1}$ approx. Obviously, the geometry of the motions carried out by mH is not fully describable by a rigid rotor in a three-fold potential. The mH motions lead to almost a complete decay of EISF at high *Q*-values.

Now let us focus on the parameters describing the distribution function of rotational times. As expected for a localized motion, the characteristic time $\tau_{o}^{MG}$ is *Q*-independent (see Figure 9b). The width of the distribution function σ also displays an approximately constant value for $Q\;<2\;{Å}^{-1}$ but a slight tendency to increase is observed at higher *Q*-values. We have thus considered the average value of σ in the low-*Q* region as that representing the actual width of the distribution of rotational times at a given temperature (lines in Figure 9a). The results corresponding to σ are inversely proportional to temperature, implying that the data are compatible with an underlying Gaussian distribution of activation energies $E_{a}^{MG}$ (see SM). The value obtained for $\sigma_{E}$ is 38.5 meV (447 K). The average activation energy $\langle E_{a}^{MG}\rangle$ is obtained from the Arrhenius fit of $\tau_{o}^{MG}$,

(17) $$\tau_{o}^{MG}=\tau_{\infty }\exp\left(\frac{\langle E_{a}^{MG}\rangle }{K_{B}T}\right)$$

leading to $\langle E_{a}^{MG}\rangle$ = 0.21 eV (2452 K) and $\tau_{\infty }$ = 0.08 ps. Compared with other glass-forming systems [41], PIB shows a very large value of $\langle E_{a}^{MG}\rangle$ (typical values are around 1000 K) but comparable to that displayed by methyl groups located in bisphenol-A moeties (like in polycarbonate, polysulfone and phenoxy [41]).

**Figure 10:**
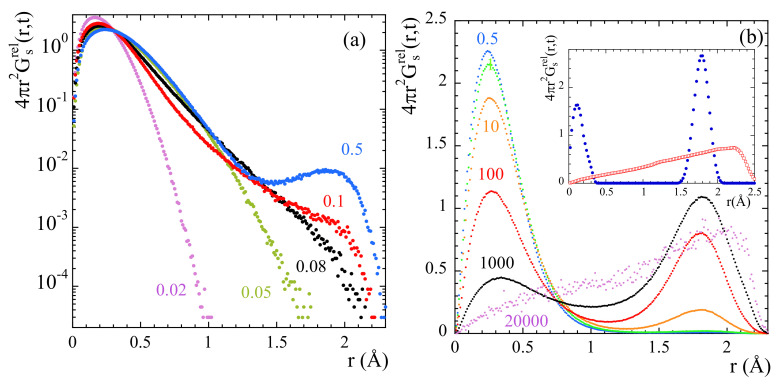
Radial probability distribution function of mH atoms relative to mC at 335 K and different times. Results in (**a**) are plotted in a logarithmic scale to enhance the subtle differences occurring at the short times shown. Inset in (**b**): Radial probability distribution function of mH atoms relative to mC calculated for a (semi-)rigid rotor (blue dots) and the same rotor with varying direction of the axis (red squares) (see text).

The direct insight of the atomic motions in real space facilitated from the simulations can help to unveil more details of mH dynamics. Figure 10 shows the radial probability distribution function calculated from the mH trajectories relative to those of mC. At times below ≈1 ps the probability shows only one clear maximum broadened by vibrational motions. With increasing time a second maximum is clearly developed which position is separated from that of the first one by the distance $r_{HH}\;=\;1.78\;Å$. The relative populations of the two peaks continuously change with increasing time. At the longest times represented, the radial probability distribution function continuously increases with distance in the physically accessible spatial range. This behavior is qualitatively different from that expected for the 3-fold model of a rigid rotor.

This observation, together with the previously commented increase of σ and decrease of EISF detected at high *Q*-values could be attributed to a superimposed translational component of mH atoms. In order to proof this hypothesis, we generated some random distributions of the accessible positions of such a proton (accordingly to the geometric configuration of a methyl group) allowing for some minor fluctuations for the methyl carbon-proton bond length and also for some stochastic vibrations around the three minima of the methyl rotor. The result is depicted by the filled circles in the insert of Figure 10 that is the one to be expected for a (semi-)rigid rotor of the corresponding geometry with a symmetry axis which direction is fixed in space. Now, if one releases this constraint and allows the axis of the rotor to vary its direction in space, the curve with empty symbols is obtained, which is very similar to those obtained in Figure 10b for the longest time.

**Figure 11:**
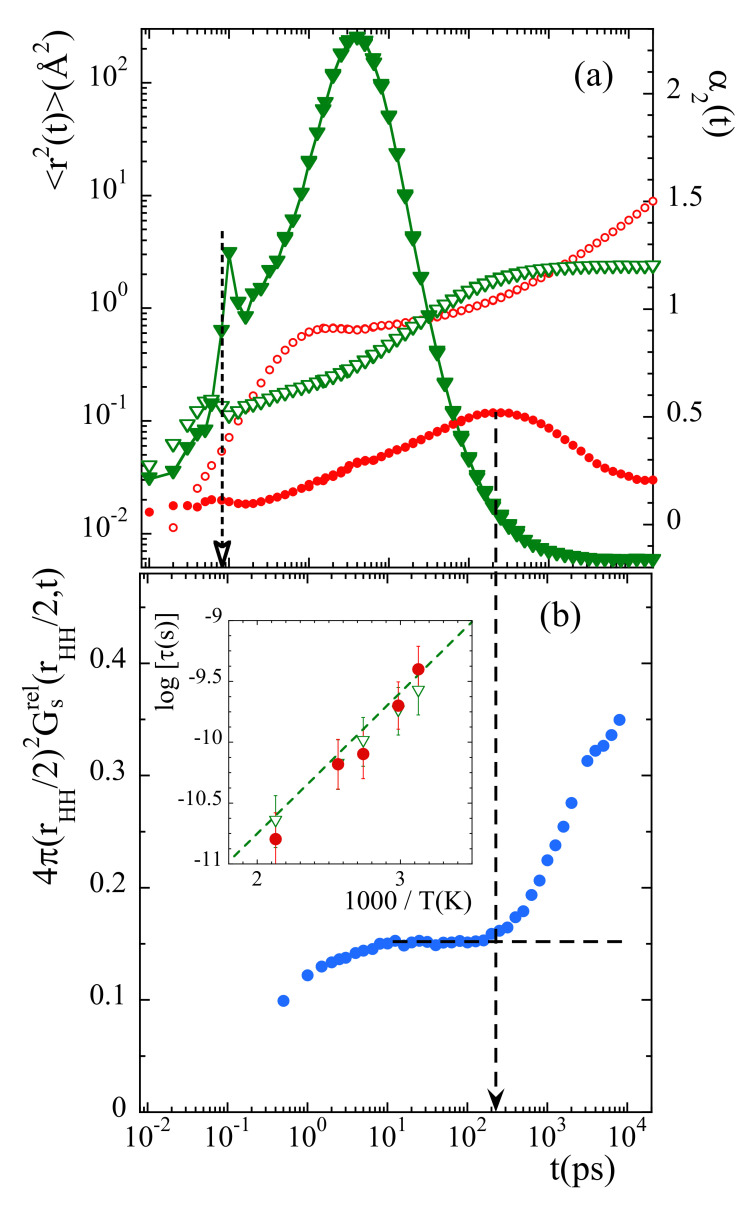
(**a**) Mean squared displacement (empty symbols) and non-Gaussian parameter (filled symbols) of main-chain carbons (circles) and relative motions of methyl-group hydrogens with respect to the methyl-group carbon (triangles). Vertical dotted arrow marks the value of $\tau_{\infty }$ in Equation (17). (**b**): Time dependence of the value of the function $4\pi r^{2}G_{s}^{rel}\left(r,t\right)$ evaluated at $r=r_{HH}/2$. The horizontal dashed line shows the expected asymptotic behavior in the stationary state of rotations, and the vertical dashed arrow the time at which the data depart from such a behavior. All results correspond to 335 K. Insert: Arrhenius plot compiling the average residence time for methyl-group rotational motions $\tau_{R}^{o}$ (empty triangles), the characteristic time for the maximum of the non-Gaussian parameter $t^{⋆}$ corresponding to main-chain carbons (filled circles) and the δ-process reported from NMR [36] and ESR [37] investigations (dashed line).

To determine the time at which the probability distribution function $4\pi r^{2}G_{s}^{rel}\left(r,t\right)$ deviates from the expected behavior for a rotor with fixed axis, we have considered the value of this function at $r\;=\;r_{HH}/2\;=\;0.89\;Å$. This parameter represents the way how the minimum in the probability distribution function is getting populated. In principle, its value should remain constant in time once the stationary regime for rotations has been achieved. Figure 11b shows the results obtained at 335 K. The time at which deviations start is about 200 ps. For comparison, Figure 11a shows with circles the mean squared displacement $\langle r^{2}\left(t\right)\rangle$ and the non-Gaussian parameter $\alpha_{2}\left(t\right)$ calculated for main-chain carbons at the same temperature. A characteristic time for the processes developed in the cageing regime $t^{⋆}$ is usually defined as that where the $\alpha_{2}$-parameter reaches its maximum. For main-chain carbons, $t^{⋆}$ is very close to 200 ps at this temperature. Thus, the non-Gaussian events undergone by backbone atoms seem to be at the origin of the peculiarities observed for methyl-group rotations.

The inverted triangles in Figure 11a show the results corresponding to the relative motion of mH with respect to mC at 335 K. At this temperature, for t≥ 1 ns mH explore the full available space around mC. There, the function $F_{s}^{rel}\left(Q,t\right)$ reaches the limit t→∞ (see Figure 8b). From the comparison between the mean squared displacements in Figure 11a we can see that the development of the localized motions by the methyl groups’ hydrogens takes place just during the occurrence of the decaging events in the backbone atoms. At longer times, in the region where $\langle r_{rel}^{2}\left(t\right)\rangle$ is constant, $\langle r_{cC}^{2}\left(t\right)\rangle$ follows the sublinear increase $\langle r^{2}\left(t\right)\rangle \;\propto \;t^{\beta }$ characteristic for the α-relaxation.

We can now consider the information provided by the distribution functions of rotational times for mH, H(logτ). From these functions, the values of the average residence time for methyl-group hydrogens $\tau_{R}^{o}$ can be obtained for the different temperatures (since $\tau_{R}$ is related with τ through $\tau_{R}\;=\;3\tau /2$, see SM, $\tau_{R}^{o}\;=\;3\tau_{o}/2$). This time is represented in the insert of Figure 11b together with the characteristic time for decaging processes of backbone carbons $t_{cC}^{⋆}$. Both times almost perfectly agree for all temperatures. Thus, methyl-group rotations and main-chain decaging are clearly coupled for PIB in the temperature range investigated. A similar conclusion was deduced from the MD-simulations reported in Ref. [38]. Finally, in the same figure we have also included (dashed line) the characteristic times reported for the so-called δ-relaxation from NMR [36,38] and ESR [37] investigations. The δ-process had been assigned by Slichter [36] to originate undoubtedly from a methyl group rotation. The excellent agreement found between our results and this process definitely supports our analysis.

Furthermore, interestingly, we note that at short times (around 0.08 ps) the $\langle r^{2}\left(t\right)\rangle$ and $\alpha_{2}\left(t\right)$ functions calculated for the relative motions of mH with respect to mC display marked peaks suggesting a kind of ’recoil’ effect (see Figure 11a). This feature occurs just when the second maximum in the $4\pi r^{2}G_{s}^{rel}\left(r,t\right)$ function starts to be populated (see Figure 10a), coinciding with the value of the prefactor $\tau_{\infty }$ in Equation (17).

**Figure 12:**
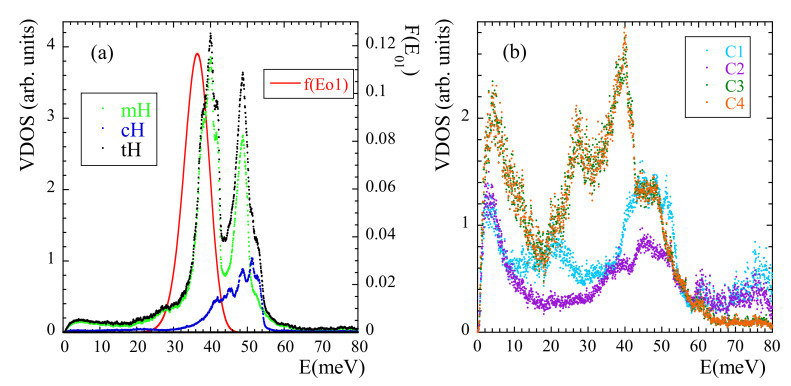
Vibrational density of states calculated at 25 K for mH, cH and tH (**a**) and for the different carbons [C1: methylene cC; C2: quaternary cC; C3 and C4: mC] (**b**). The line in (**a**) represents the librational peak $F(E_{01})$ deduced within the 3-fold approximation from the distribution of activation energies obtained from the analysis of the functions $F_{s}^{rel}\left(Q,t\right)$ at high temperatures.

We now address another manifestation of methyl-group dynamics in NS experiments, namely the presence of librational peaks in the inelastic spectra. A peak in the VDOS reveals the energy of the transition between the ground and first excited level $E_{01}$ (see SM). We have calculated the VDOS corresponding to mH, cH and tH [see Figure 12a]. In this way, the ’selective deuteration’ invoked by Adams et al. [42] was realized. In the region where the NS experiments showed the broad peak for tH we can clearly distinguish two peaks. The first (and mean) peak indeed corresponds to a mH peak (presumably that corresponding to $E_{01}$). The second peak of the tH-VDOS at about 50 meV, which in the experiments was not really resolved from the first one, is also mainly due to a pronounced peak in the VDOS of mH, but contains additional cH-contributions in the range 40–55 meV. We note that a double-peak structure in the range ≈34–44 meV was also observed in the VDOS of polysulfone, polycarbonate and phenoxy [41]. Thus, there are striking similarities in the behavior of PIB and those systems, regarding both, the presence of a double peak in this energy region and the above commented unusually large value of $\langle E_{a}^{MG}\rangle$ (and accordingly, of $\langle E_{01}\rangle$).

The VDOS of the carbons in the system has also been calculated, distinguishing even the four different carbons. The peak at about 40 meV in the VDOS of mH appears as main signature of the VDOS in mC. A peak at about 50 meV is present in the VDOS of all the carbon species, and is specially pronounced in the methylene cC. Thus, the appearance of a second peak in the VDOS of mH at about 50 meV seems to be a consequence of the strong coupling of mH and cC dynamics, as proposed by Adams et al. [42] and is consistent with our conclusions from the analysis of the rotational dynamics of mH at high temperature.

As pointed out in Ref. [42], the peak attributed to methyl-group librations is rather broad. In principle, such broadening could be understood as consequence of the disorder in the glassy material. In fact, this ingredient has already been taken into account analysis of the $F_{s}^{rel}\left(Q,t\right)$ functions by invoking the RRDM. From the obtained distribution of activation energies $f(E_{a}^{MG})$ and using the proper relationships (see SM) the corresponding distribution function of librational energies $F(E_{01})$ can be obtained under the assumption of an underlying 3-fold potential. The result is included in Figure 12a. Though the deduced average value $\langle E_{01}\rangle \;=$ 36 meV is slightly shifted toward lower energies with respect to the simulated one, the broadening is well reproduced. The discrepancy observed for $\langle E_{01}\rangle$ could be due to the fact that the analysis of $F_{s}^{rel}\left(Q,t\right)$ was made for temperatures above $T_{g}$. There, the potential ’seen’ by the methyl groups could be slightly different from that in the glassy state, due to the softening of the non-bonded interactions at high temperature.

### 4.3. Revisiting Neutron Scattering Studies

As it was mentioned in the Introduction, to shed light on the origin of the dielectric β-process, in Ref. [34] backscattering NS-measurements were performed on fully protonated PIB in the temperature range 260–280 K. There, the α-relaxation was not expected to sensitively contribute in the experimental window accessed. Even so, its contribution was considered in the data analysis assuming the merging of α and β-relaxations as statistically independent processes (i.e., the corresponding intermediate scattering functions are combined as a simple product). The β-process was represented by a localized motion involving atomic jumps between two equivalent positions separated by a distance *d*. The corresponding EISF is then given by

(18) $$EISF=\frac{1}{2}\left(1+\frac{\sin(Qd)}{Qd}\right).$$

The resulting value obtained for *d* was d=2.7Å. If protonic jumps over such large distances actually occurred in PIB, we could have a chance to identify them in the radial probability distribution function. The best conditions would be at the lowest temperature investigated (320 K), where we could expect to have the least contribution from the diffusional motions involved in the α-process. According to the dielectric results, at 320 K the occurrence of jumps should manifest at most at about 1 ns (where the distribution function of the corresponding characteristic times reaches its maximum). As can be seen in the insert of Figure 6a, at such time the function $4\pi r^{2}G_{s}^{tH}\left(r,t\right)$ seems indeed to show a kind of double-peak structure, but the location of the peak (nearly a hump, see arrow in the figure) centered at larger distances would be around, at most, 2 Å. In fact, the inspection of this function for mH and cH separately reveals that the cH atoms do not show any clear hint of jumps. Conversely, we have successfully characterized the localized motions of mH as methyl-group rotations. These are the events producing the second feature at ≈2Å in the distribution function of tH.

It is worth or remark that the result of an apparently large motional amplitude for localized motions arises as a consequence of the deconvolution procedure assuming statistically independence of localized and diffusive-like processes. To illustrate this, we may define an ‘apparent EISF’ from the values of the above defined deconvoluted function at t≈2 ns. At this time, $F_{s}^{Deconv}\left(Q,t\right)$ seems to approach a plateau at low temperatures, as shown in Figure 7a. To take into account the different vibrational dynamics of mH and cH, we have corrected the results by the value of this function at t=2 ps, $EISF_{app}\equiv F_{s}^{Deconv}\left(Q,t=2ns\right)/F_{s}^{Deconv}\left(Q,t=2ps\right)$. The resulting values are shown as circles in Figure 8a. This kind of data treatment for mH motions results in apparent EISF-values that are lower than those corresponding to a methyl-group rotation. The fit of Equation (18) to the such obtained data in the experimentally accessed *Q*-window deliver a jump distance of 2.6 Å (see solid line in Figure 8a). Thus, the coupling between methyl group rotations and main-chain dynamics in PIB invalidates for this polymer the hypothesis of diffusive and localized motions being statistically independent processes.

## 5. Conclusions

In this work we have shown the power of properly validated fully atomistic molecular dynamics simulations to unravel the details of the atomic motions at inter- and intra-molecular length scales in polymers. First of all, the realism of the simulated cell has been proved by direct comparison of magnitudes calculated from the simulated atomic trajectories and determined on the real polymer by neutron scattering experiments. Taking advantage of the information available from the atomic trajectories, the simulations have been exploited to disentangle the self-motions of the different kinds of nuclei in PIB. The different atomic species display distinct motions, being rotations of methyl groups coupled with main-chain dynamics; in all cases, a crossover from Gaussian to non-Gaussian behavior is observed. In more detail, we have shown that:

- The motions of the different atomic species are distinct. All atoms manifest Gaussian-like behavior at low *Q*-values and deviations from it at high *Q*s.
- The anomalous jump diffusion model captures this behavior by invoking an underlying distribution of elementary jumps at the origin of the sublinear diffusion of atoms in the α-relaxation regime. The typical lengths of the jumps deduced are ≈0.4Å for main-chain atoms, in accordance with results reported for other polymers.
- Due to rotations, methyl-group hydrogens display an enhanced mobility in the region where the other atoms show decaging processes. These motions are manifested in low values of the shape parameter and shorter characteristic times at small length scales.
- The usually invoked statistically independence of rotation and translational motions does not work in polyisobutylene. The methyl-group rotations could be fully characterized by analyzing the relative motion of these atoms with respect to the carbon at which the are linked. The rotational rate distribution model gives account for these motions, though deviations are found at high *Q*-values. They can be rationalized as due to non-Gaussian processes taking place during the cageing regime of backbone atoms, which allow a superimposed motion of the rotation axis.
- The methyl-group rotations are coupled with the main-chain dynamics, as deduced from the coincidence of the characteristic times for rotations and non-Gaussian events within the cage of main-chain carbons. This coupling was also concluded from a study combining MD-simulations and NMR experiments [38] and has also been deduced from the analysis of the coherent structure factor of the present simulations [55]. This shall be the origin of the high value observed for the average potential barrier for methyl-group dynamics, leading consequently to a high value of the librational frequency. In fact, a double peak appears in the VDOS of methyl-group hydrogens, where the peak at higher energies occurs where main-chain carbons also present a characteristic peak.
- The information provided by the simulations in real space rules out the occurrence of large amplitude jumps (≈3Å) associated to the β-process, as proposed from the analysis of neutron scattering data on protonated samples [34]. The deduction of such a motion is an artifact arising when the hypothesis of statistically independence of α-relaxation and localized processes is assumed. The reason is the coupling of methyl-group rotations and main-chain dynamics, possibility that was already suggested by the authors of Ref. [34] as an explanation of the apparently incompatible observations from incoherent and coherent scattering studies.

## Figures and Tables

**Table 1:** Values of the preferred jump length $\ell_{o}$ and activation energy of the characteristic time $\tau_{o}$, $E_{o}^{AJD}$, involved in the description by anomalous jump diffusion model, for the different atomic species distinguished in PIB.

Atomic Species	$\ell_{o}$(Å)	$E_{o}^{\mathrm{AJD}}$(eV)
cC	0.32 ± 0.06	0.44 ± 0.13
mC	0.54 ± 0.03	0.47 ± 0.11
cH	0.41 ± 0.03	0.47 ± 0.13
mH	0.50 ± 0.03	0.32 ± 0.10
tH	0.57 ± 0.03	0.42 ± 0.13

## Data Availability

The data presented in this study are openly available in: https://ehubox.ehu.eus/s/6zpwz274ntyw5tt.

## Supplementary Materials

The following are available online at https://www.mdpi.com/2073-4360/13/4/670/s1.

## Abbreviations

The following abbreviations are used in this manuscript:

NS	Neutron scattering
PVME	poly(vinyl methyl ether)
PH	Phenoxy
PVC	Poly(vinyl chloride)
PB	1,4-Polybutadiene
PVE	Poly(vinyl ethylene)
PI	Polyisoprene
PIB	Polyisobutylene
NSE	Neutron spin echo
MD	Molecular dynamics
PMMA	Poly(methyl metacrylate)
PEP	Poly(ethylene propylene)
hh-PP	head-to-head Polypropylene
PVAc	Poly(vinyl acetate)
PVP	Poly(vinylpyrrolidone)
PEMA	Poly(ethyl methacrylate)
PTHF	Poly(tetrahydrofurane)
VDOS	vibrational density of states
cC	main-chain carbons
cH	main-chain hydrogens
mC	methyl-group carbons
mH	methyl-group hydrogens
tH	total hydrogens
SBR	styrene-butadiene-rubber
PS	polystyrene
