# Supplementary material for: Disentangling Self-Atomic Motions in Polyisobutylene by Molecular Dynamics Simulations
Source: Polymers (Basel). 2021 Feb 23;13(4):670. doi: 10.3390/polym13040670 (PMC7927061; doi:10.3390/polym13040670)
Supplement: Supplementary file 1 [file polymers-13-00670-s001.pdf]

Disentangling Self-Atomic Motions in Polyisobutylene by Molecular Dynamics Simulations. Supplemental Material

Yasmin Khairy,[1,2] Fernando Alvarez,[2,3,4] Arantxa Arbe,[2,3] and Juan Colmenero[2,3,4,5,*]

[1]*Physics Department, Faculty of Science, Zagazig University, 44519, Zagazig, Egypt*

[2]*Centro de Física de Materiales (CFM) (CSIC–UPV/EHU), Paseo Manuel de Lardizabal 5, 20018 San Sebastián, Spain*

[3]*Materials Physics Center (MPC), Paseo Manuel de Lardizabal 5, 20018 San Sebastián, Spain*

[4]*Departamento de Polímeros y Materiales Avanzados: Física, Química y Tecnología (UPV/EHU), Apartado 1072, E-20080 San Sebastián, Spain*

[5]*Donostia International Physics Center, Paseo Manuel de Lardizabal 4, 20018 San Sebastián, Spain*

[*] juan.colmenero@ehu.eus

I. ANOMALOUS JUMP DIFFUSION MODEL: RESULTS ON THE JUMP LENGTH

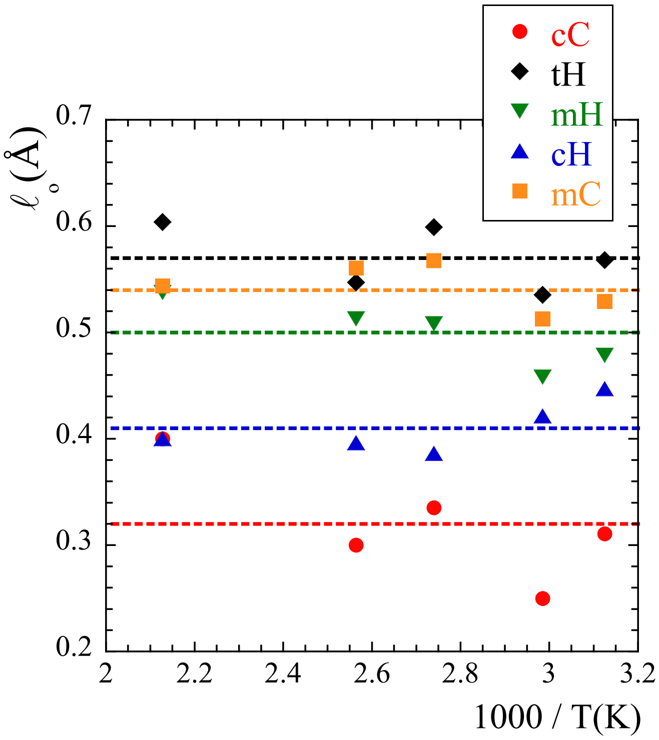

FIG. S1. Temperature dependence of the preferred jump length involved in the anomalous jump diffusion model for the different kinds of atoms in PIB. Errors are about 0.1. Dotted lines show the mean values for the different species.

II. CHECKING THE DECONVOLUTION APPROACH FOR METHYL-GROUP HYDROGENS

In the paper we show that the deconvolution approach clearly fails at long times. We have also checked whether it works in the time region where methyl-group rotations seem to occur (between ≈ 2 ps and 2 ns) for 335 K, see Fig. 7(a) in the article. We have thus tried to describe the deconvoluted function obtained as $F_s^{Deconv}(Q,t) = F_s^{mH}(Q,t)/F_s^{cH}(Q,t)$ at 335 K in the time interval 2 ps $\leq t \leq$ 2 ns in terms of the rotational rate distribution model (RRDM) [1–3] described in the next section. By considering expressions S-5 and S-6, Eq. S-13 was used to fit the data. The width σ and the center τ_o^{MG} of the log-normal distribution function of characteristic times $H(\log\tau)$ are the only free parameters (in addition, obviously, to an amplitude A accounting for the microscopic contribution). It turned out that the fit at low Q-values ($Q < 1.5$ Å^{-1}) was impossible by fixing the geometry of the motion as that expected for methyl-group rotations. Leaving EISF as free parameter a reasonable description was achieved. The obtained values for the EISF are depicted in Fig. S2 as empty squares. In the Q-range $Q < 1.5$ Å^{-1} (corresponding to large length scales) they are sensitively lower than the theoretical ones. In this Q-range, the results regarding the other parameters involved in the RRDM –σ and τ_o^{MG}– are also inconsistent with the expectations (Q-independent values), as can be seen in Fig. S3 (empty squares).

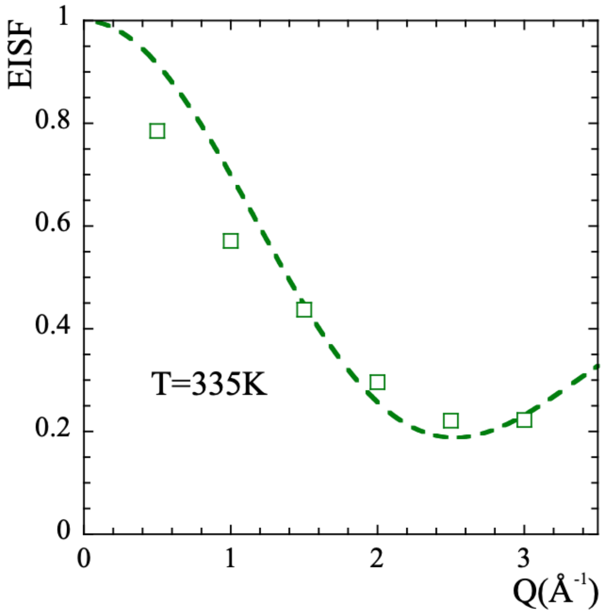

FIG. S2. EISF values obtained from the application of the RRDM to the resulting function from the deconvolution approach in the time interval 2 ps $\leq$ t $\leq$ 2 ns. The dotted line is the theoretical prediction (Eq. S-6).

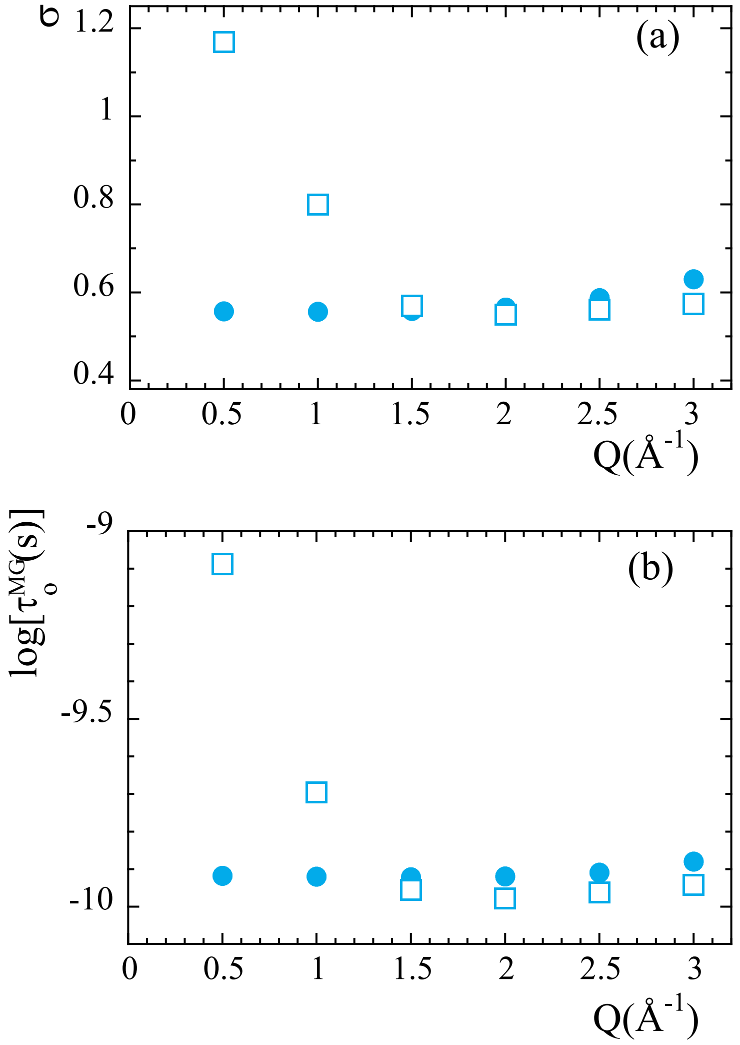

FIG. S3. Momentum transfer dependence of the RRDM parameters σ (a) and τ_o^{MG} (b) obtained at 335 K from the fit of $F_s^{Deconv}(Q,t)$ (empty squares) and $F_s^{rel}(Q,t)$ (solid circles).

III. METHYL GROUP DYNAMICS: THE ROTATIONAL RATE DISTRIBUTION MODEL

This section presents the elementary theoretical background used to treat the problem of methyl group dynamics in the manuscript. Further and more general information about methyl-group dynamics in glass-forming systems can be found e. g. in Ref. [3].

The simplest model for methyl group rotations considers that only the first term (V_3) is relevant in the Fourier series in which the potential $V(\phi)$ can be expanded (3-fold approximation):

$$V(\phi) = \sum_{n=1}^{\infty} \frac{V_{3n}}{2} \left[1 - \cos(3n\phi + \delta_{3n})\right]. \tag{S-1}$$

Here ϕ is one characteristic coordinate, which is measured in the plane perpendicular to the C_3-symmetry axis of the methyl group. By solving the corresponding Schrödinger equation, one can obtain the quantized energy levels of a threefold potential. The energy levels E_{0i}, i=0, 1, 2, ... corresponding to the individual potential wells are named torsional or librational levels. Due to the coupling between the single-well wave functions, these energy levels are split. Three relevant magnitudes –which are experimentally accessible by neutron scattering– can be defined from the methyl group rotational potential: (1) the transition energies between the librational levels, mainly the transition E_{01} between the ground state and the first excited state. (2) The splitting energy of the ground librational state ($\Delta_0 = \hbar\omega_t$) characterized by the quantum 'tunneling frequency' ω_t. (3) The activation energy E_a^{MG}, related to the methyl group 'classical hopping' between adjacent wells. This energy is defined as the difference between the top of the barrier and the ground state. These three quantities are direct functions of the potential barrier V_3:

$$E_{01}(meV) = 0.470 V_3^{0.548} \tag{S-2}$$

$$\hbar\omega_t(meV) = 0.655\left(1 + \frac{V_3}{2.67}\right)^{1.06} \exp\left[-\left(\frac{V_3}{4}\right)^{0.5}\right] \tag{S-3}$$

$$E_a^{MG}(K) = 0.598 V_3^{1.05} \tag{S-4}$$

(V_3 expressed in K).

In the low temperature regime (a few K), quantum effects dominate the dynamics, and tunneling transitions between the split librational levels are expected. At high temperature, the methyl group dynamics can be described by hopping processes over the potential barriers. We will focus on the latter case, which is that relevant in this study. Assuming that the hopping time between two equilibrium positions is negligible in comparison with the residence time τ_R between consecutive jumps in a 3-fold potential, the characteristic time is $\tau = 2\tau_R/3$ and the intermediate incoherent scattering function for a methyl group can be written as:

$$F_s^\tau(Q,t) = \mathrm{EISF} + (1 - \mathrm{EISF}) \exp\left(-\frac{\mathrm{t}}{\tau}\right). \tag{S-5}$$

The elastic incoherent structure factor EISF carrying the information about the geometry of the motion in the 3-fold approximation is given by:

$$EISF = \frac{1}{3}\left(1 + 2\frac{\sin(Qr_{HH})}{Qr_{HH}}\right). \tag{S-6}$$

Here $r_{HH} = 1.78$ Å is the distance between the hydrogens in the methyl group. The temperature dependence of τ is determined by the activation energy E_a^{MG} by the Arrhenius law, $\tau = \tau_\infty \exp[E_a^{MG}/(K_B T)]$.

It is well known that this simple model does not work in glassy systems where the inherent disorder leads to distributions of mobilities. This aspect was introduced by the so-called rotation rate distribution model (RRDM) [1–3]. The RRDM model is based on the idea that due to the structural disorder inherent to the glassy state, there exists a distribution of potential barriers $g(V_3)$. This is assumed to be Gaussian:

$$g(V_3) = \frac{1}{\sqrt{2\pi}\sigma_V} \exp\left[-\frac{(V_3 - \langle V_3\rangle)^2}{2\sigma_V^2}\right]. \tag{S-7}$$

Here $\langle V_3\rangle$ is the average barrier and σ_V is the standard deviation of the distribution. As a consequence, the three relevant magnitudes E_{01}, $\hbar\omega_t$ and E_a^{MG} are obviously also distributed according to the corresponding distribution functions $F(E_{01})$, $h(\hbar\omega_t)$ and $f(E_a^{MG})$. The functional relations (Eqs. S-2,S-3,S-4) between E_{01}, $\hbar\omega_t$ and E_a^{MG} and V_3 allow for a straightforward transformation between $g(V_3)$ and $F(E_{01})$, $h(\hbar\omega_t)$ and $f(E_a^{MG})$:

$$g(V_3)dV_3 = F(E_{01})dE_{01} = -h(\hbar\omega_t)d(\hbar\omega_t) = f(E_a^{MG})dE_a^{MG}. \tag{S-8}$$

Given the almost linear relationship between E_a^{MG} and V_3 (Eq. S-4), a Gaussian functional form can be considered as a good approximation for the associated distribution of activation energies $f(E_a^{MG})$:

$$f(E_a^{MG}) = g(V_3)\frac{dV_3}{dE_a^{MG}} = \frac{1}{\sqrt{2\pi}\sigma_E} \exp\left[-\frac{\left(E_a^{MG} - \langle E_a^{MG}\rangle\right)^2}{2\sigma_E^2}\right] \tag{S-9}$$

with average energy $\langle E_a^{MG}\rangle$ and standard deviation σ_E. The preexponential factor τ_∞ is assumed to be independent of the barrier, leading to a log-Gaussian distribution of characteristic times for classical hopping:

$$H(\log\tau) = \frac{1}{\sqrt{2\pi}\sigma} \exp\left[-\frac{\left(\log\tau - \log\tau_o^{MG}\right)^2}{2\sigma^2}\right]. \tag{S-10}$$

where

$$\sigma = \frac{\sigma_E \log(e)}{K_B T} \tag{S-11}$$

is the width of the distribution $H(\log\tau)$ centered at τ_o^{MG}, which is the characteristic time corresponding to the average activation energy:

$$\tau_o^{MG} = \tau_\infty \exp\left(\frac{\langle E_a^{MG}\rangle}{K_B T}\right) \tag{S-12}$$

Thus, the final scattering function is built by adding the scattering functions of the hydrogens located in the different environments weighted by the distribution function,

$$F_s^{rot}(Q,t) = \int_{-\infty}^{+\infty} H(\log\tau) F_s^{\tau}(Q,t) d(\log\tau). \tag{S-13}$$

[1] A. Chahid, A. Alegría, and J. Colmenero, Macromolecules **27**, 3282 (1994).

[2] J. Colmenero, R. Mukhopadhyay, A. Alegría, and B. Frick, Phys. Rev. Lett. **80**, 2350 (1998).

[3] J. Colmenero, A. J. Moreno, and A. Alegría, Progress in Polymer Science **30**, 1147 (2005).
